# Ethyl acetate produced by *Hanseniaspora uvarum* is a potential biocontrol agent against tomato fruit rot caused by *Phytophthora nicotianae*

**DOI:** 10.3389/fmicb.2022.978920

**Published:** 2022-08-10

**Authors:** Ziyu Liu, Junjie Tian, Hao Yan, Delong Li, Xue Wang, Wenxing Liang, Guangyuan Wang

**Affiliations:** ^1^Shandong Province Key Laboratory of Applied Mycology, College of Life Sciences, Qingdao Agricultural University, Qingdao, China; ^2^The Key Laboratory of Integrated Crop Pest Management of Shandong Province, College of Plant Health and Medicine, Qingdao Agricultural University, Qingdao, China; ^3^Yantai Agricultural Technology Extension Center, Yantai, China

**Keywords:** postharvest fruit rot, ethyl acetate, VOCs, *Hanseniaspora uvarum*, *Phytophthora nicotianae*

## Abstract

In this study, an oomycete strain FQ01 of *Phytophthora nicotianae*, which could cause destructive postharvest disease, was isolated. At present, chemical fungicides are the main reagents used for controlling *Phytophthora* diseases. It is necessary to find new control techniques that are environmentally friendly. The biocontrol activity of *Hanseniaspora uvarum* MP1861 against *P. nicotianae* FQ01 was therefore investigated. Our results revealed that the volatile organic compounds (VOCs) released by the yeast strain MP1861 could inhibit the development of *P. nicotianae* FQ01. The major component of the VOCs produced by the yeast strain MP1861 was identified to be ethyl acetate (70.8%). Biocontrol experiments showed that *Phytophthora* disease in tomato fruit could be reduced by 95.8% after the yeast VOCs treatment. Furthermore, ethyl acetate inhibited the mycelial growth of the oomycete strain FQ01, and damaged the pathogen cell membrane. This paper describes the pioneering utilization of the yeast strain MP1861 for biocontrol of postharvest fruit rot in tomato caused by *P. nicotianae*.

## Introduction

*Phytophthora* is a genus of oomycete plant pathogens that are notorious for their devastating effects on crops, vegetables, pasture plants and trees. The *Phytophthora* complex has been found to include ~120 species (Panabieres et al., 2016). *Phytophthora nicotianae*, first isolated from tobacco, is an important pathogen because it causes severe damage to a particularly large number of host plants (Panabieres et al., 2016). More than 255 plant species can be infected by *P. nicotianae*, including tomato, tobacco, potato, citrus, and eggplant (Chowdappa et al., 2016; Panabieres et al., 2016). At present, the application of fungicides, such as formanyl, hymexazol, and dimethomorph, is the main method to prevent the occurrence of plant diseases caused by *P. nicotianae* (Han et al., 2019). However, systematic use of chemicals can lead to pathogen resistance to fungicides and pesticide residues, which in turn pollute environmental ecosystems and endanger human health and livestock. Therefore, to effectively prevent agricultural losses caused by *P. nicotianae*, it is necessary to find new ways to reduce the use of and replace fungicides.

In recent years, microbes acting as agents for the biological control of plant diseases have been recognized as among the most promising alternatives to fungicides because of their effectiveness and environmental friendliness. To date, many antagonistic bacteria and fungi, such as *Bacillus atrophaeus* (Rajaofera et al., 2018), *Pseudomonas fluorescens* (Sukhada et al., 2011), *Trichoderma* spp. (Bae et al., 2016), and *Glomus mosseae* (Sukhada et al., 2011), have been reported as biocontrol agents against *P. nicotianae*. However, the utilization of antagonistic yeasts to manage plant diseases caused by *P. nicotianae* has rarely been reported.

Antagonistic yeasts have many advantages, such as high safety, wide antimicrobial spectrum, strong stress resistance, genetic stability, cultivability, and low nutrient requirements (Cai et al., 2015). Many plant diseases have been found to be controlled by antagonistic yeasts. For example, S*poridiobolus pararoseus* could protect table grape from decay caused by *Aspergillus niger* (Li et al., 2017); *Metschnikowia fructicola* antagonized the pathogen *Botrytis* causing rot in stored grapes (Kurtzman and Droby, 2001); *Aureobasidium pullulans* provided improved biocontrol against the pathogen *Geotrichum citri-aurantii* causing sour rot in citrus (Klein and Kupper, 2018); *Candida oleophila* reduced papaya anthracnose caused by *Colletotrichum gloeosporioides* (Gamagae et al., 2003). These examples clearly indicate the potential applicability of antagonistic yeasts in biological control and also highlight the limited knowledge of the control of *P. nicotianae* with such organisms. Therefore, it is of great significance to screen new antagonistic yeasts for protecting plants from *Phytophthora* infection.

*Hanseniaspora uvarum*, a kind of apiculate yeast with a lemon-shaped cell morphology, is widely distributed on the surface of mature fruit, plant leaves, fermented beverages, soils and many other environments (Guaragnella et al., 2019). Previous studies demonstrated that *H. uvarum* had potential for controlling postharvest decay of strawberry (Cai et al., 2015). The volatile organic compounds (VOCs) from *H. uvarum* had been shown to inhibit the growth of the pathogenic fungus *B. cinerea* (Wang L. Y. et al., 2019). Further study revealed that *H. uvarum* could reduce the natural decay of grape berries without impairing quality parameters (Liu et al., 2010). These findings clearly showed that *H. uvarum* has promising potential in the biological control of plant diseases.

In this study, the yeast strain MP1861 of *H. uvarum* was identified as a potential biocontrol strain against *P. nicotianae*. Evaluating the antifungal efficacy of the VOCs from the yeast strain MP1861 and analysis of the active components in the VOCs were further conducted. Ours findings promoted the application of antagonistic yeast in the control of *Phytophthora* diseases. To our knowledge, the present work represents the first report of utilization of antagonistic yeast to manage postharvest fruit disease caused by *P. nicotianae*.

## Materials and methods

### Yeast and oomycete strains

*H. uvarum* MP1861 isolated from the leaves of *Kalopanax septemlobus* and *P. nicotianae* FQ01 isolated from rotten tomato fruit were maintained on YPD (1% yeast extract, 2% peptone, 2% dextrose and 2% agar) and PDA (20% potato extract, 2% dextrose, and 2% agar) slants at 4°C, respectively.

### Assay of the pathogenicity of *P. nicotianae* FQ01

After growth on PDA plate at 26°C for 3 days, five mm diameter mycelial agar plugs of the pathogen FQ01 were transferred to tobacco leaves, tomato fruit, potato tuber, cucumber, and eggplant, respectively, followed by incubation at 26°C for 4 days. Finally, the lesions caused by the pathogen FQ01 were investigated.

### Isolating and screening the yeast strains with biocontrol activities

Plant samples, including roots, stems, leaves, fruit, and flowers, were collected from Laoshan National Forest Park in Qingdao, China. Yeast strains were isolated using the procedures as previously described (Wang et al., 2018). Briefly, five grams of plant sample was suspended in 50 ml YPD medium supplemented with 0.01% chloramphenicol and then incubated at 28°C, 180 rpm, for 2 days. The culture was diluted to 10^−6^ using sterilized water. Then, 200 μl of the diluted cell culture was evenly plated on an YPD plate and grown at 28°C for 2 d. The obtained yeast colonies were maintained at 4°C.

Antifungal activities of the obtained yeast strains were performed using the dual culture method (Rajaofera et al., 2018). In brief, the pathogen FQ01 was inoculated in the center of PDA plate, and the isolated yeasts were inoculated around the pathogen. After inoculation at 26°C for 4 d, the growth inhibitions of the pathogen FQ01 on PDA plate by different yeast strains were observed.

### Morphological characterization

After growth on YPD plates at 26°C for 48 h, the morphological characterization of the yeast strain MP1861 was performed. The oomycete strain FQ01 was first grown on PDA plates at 26°C for 4 d. Then, ~10 mycelial plugs (0.5 cm diameter) of the oomycete strain FQ01 were transferred into clarified V8 juice followed by incubation at 26°C for 48 h. After 3–4 washes using ultrapure water, the actively growing mycelia were covered by ultrapure water and incubated at room temperature for 3–7 d. Finally, the sporangia and chlamydospores produced by the oomycete strain FQ01 were recorded.

### Phylogenetic analysis of different microorganisms

DNA extraction from the oomycete strain FQ01 and the antagonistic yeast MP1861 were carried out with Fungi Genomic DNA Purification Kit (Sangon Biotech, China) according to the manufacturer's instructions, respectively. ITS region of the oomycete strain FQ01 was amplified using PCR with the universal fungal primers ITS1 and ITS4 (Garcia-Estrada et al., 2020). D1/D2 26S rDNA region of the antagonistic yeast MP1861 was amplified using PCR with the primers NL1 and NL4 (Wang et al., 2018). The generated fragments were sequenced. The obtained sequences were blasted using BLASTN. Phylogenetic tree based on a neighbor-joining analysis was constructed using MEGA 4.0 (Tamura et al., 2007).

### Pathogen inhibition by the VOCs emitted from the antagonistic yeast

The mycelial inhibition by the VOCs emitted from the yeast strain MP1861 was performed in closed Petri dishes according to previously methods (Huang et al., 2011) with appropriate modifications. In brief, the pathogen FQ01 was incubated on PDA plate at 26°C for 3 days. Then, a mycelial agar plug (0.5 cm diameter) from the colony margin of *P. nicotianae* FQ01 was transferred onto one PDA plate (6.0 cm diameter). Meanwhile, the other PDA plate was inoculated with 100 μl yeast cells (3.0 × 10^4^ cells/mL). The two plates were then sealed with parafilm. Subsequently, the sealed plates were placed at 26°C for 4 d. The colony diameter of *P. nicotianae* FQ01 in each plate was measured. The inhibition of mycelial growth by the VOCs produced by the yeast strain MP1861 was calculated according to the formula [(D_ck_ – D_VOCs_) / D_ck_] × 100 %, where D_ck_ and D_VOCs_ indicate the average diameter of *P. nicotianae* colonies grown on PDA without or with the VOCs treatment, respectively.

### GC/MS analysis

VOCs were first adsorbed by solid-phase microextraction (SPME) (Supelco, 57329-U). The VOCs adsorbed above were then analyzed by a TSQ8000 mass spectrometer (Thermo Fisher). Briefly, a fused-silica fiber coated with divinylbenzene (DVB), carboxen (CAR), and polydimethylsiloxane (PDMS) was traversed into the sampling headspace bottle *via* a silicone septum. After adsorption at 40°C for 60 min, the fiber was inserted into a GC injector to desorb the volatile compounds at 250°C for 5 min. The released components were then separated by a TG-5SILMS GC column (Thermo Fisher, 30 m × 0.25 mm × 0.25 μm). The carrier gas of the GC was helium, and the gas flow rate was 1.3 mL per min. The temperature program in the GC oven consisted of 40°C for 2.0 min, raising to 100°C at 3°C per min and then to 240°C at 20°C per min, 240°C for 5.0 min. The electron ionization (EI) applied was fixed at 70 eV, and the ion source temperature used in the MS was set at 230°C. The m/z scan range was 40–400 amu. The results obtained above were searched against the database of the National Institute of Standards and Technology (NIST).

### Fluorescence microscopy to evaluate the toxicity of ethyl acetate to the oomycete strain FQ01

To investigate the damage caused by ethyl acetate to *P. nicotianae* FQ01, we performed a propidium iodide (PI) fluorescence assay. In brief, the hyphal cells of *P. nicotianae* FQ01 were treated with different concentrations of ethyl acetate (Sangon Biotech) from 0 to 7.18 g/L at 26°C for 24 h, respectively. Then, the treated mycelia were stained with PI fluorescent probe (1 mg/L, Solarbio) 30 min at room temperature in the dark (Jing et al., 2017; Han et al., 2019). Finally, the treated mycelia were photographed using an EVOS M5000 fluorescence imaging system (Thermo Fisher).

### Control of *Phytophthora* disease on tomato fruit by the VOCs from the yeast strain MP1861

The inhibitory assay of *Phytophthora* disease on tomato fruit by the volatiles of *H. uvarum* MP1861 was performed in double closed Petri plates. First, the surface of sixteen healthy tomato fruit (3.0–3.5 cm length and 2.0–3.0 cm diameter) were disinfected using 70% ethanol. After rinsing in sterile distilled water, the fruit was dried at room temperature on an ultra-clean workbench. Each fruit was poked once with a toothpick, followed by inoculation with one mycelial plug (0.5 cm diameter) of the oomycete strain FQ01. The treated fruit were arranged on a glass Petri plate (3.0 cm height and 15.0 cm diameter). Meanwhile, the other Petri plate containing PDA medium was inoculated with 200 μl yeast cell suspension (1.0 × 10^9^ cells/mL) on the medium surface (Qin et al., 2017). An equal amount of sterile distilled water was used as the control. The two glass Petri dishes were then sealed with parafilm. The closed Petri plates were incubated at 26°C for 3 d. Finally, the lesions on tomato fruit and the ethyl acetate production in the closed Petri plates were investigated. There were three replicate trials in each test. The inhibition rate of the VOCs on tomato fruit disease was calculated by equation [(L_ck_ – L_VOCs_) / L_ck_] × 100%. L_ck_ and L_VOCs_ indicate the average area of lesions infected by *P. nicotianae* FQ01 on tomato fruit without or with the VOCs treatment, respectively.

### Statistical analysis

Data represent the mean values ± standard deviation (SD). The obtained data were calculated by the one-way ANOVA for statistical tests. A value of *p* < 0.05 was considered to be statistically significant.

## Results

### Identification and pathogenicity investigation of the pathogen FQ01

**Figure 1:**
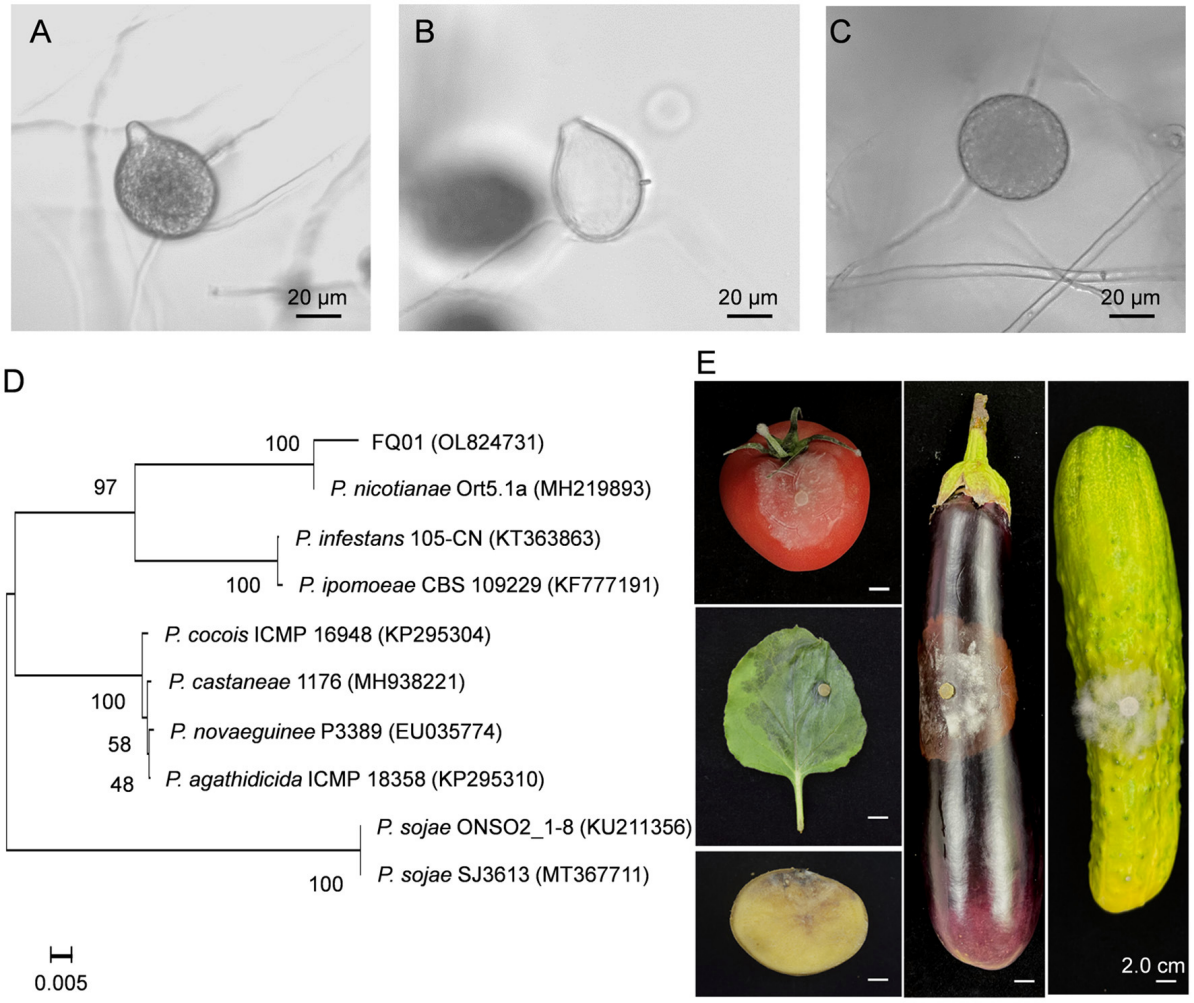
Identification of the pathogen FQ01. **(A)** Sporangial morphology before the release of zoospores. **(B)** Sporangial shell after the release of zoospores. **(C)** Chlamydospore morphology. **(D)** Phylogenetic tree of the pathogen FQ01 and other *Phytophthora* strains based on a neighbor-joining analysis of ITS sequence. **(E)** Symptoms of plant diseases caused by *P. nicotianae* FQ01 infection.

The pathogen FQ01 was isolated from rotten tomato, which could cause postharvest loss of tomato fruit. The sporangia produced by the pathogen FQ01 were ovoid and obturbinate with a prominent papilla (Figures 1A,B). The pathogen FQ01 could also produce abundant spherical chlamydospores (Figure 1C). The ITS region of the pathogen FQ01 was sequenced. The obtained sequence has been stored in GenBank (accession No. OL824731). A phylogenetic tree according to the ITS region of the pathogen FQ01 was further constructed. The results revealed that the pathogen FQ01 was clustered in one branch with *P. nicotianae* (Figure 1D). These observations clearly showed that the pathogen FQ01 was *P. nicotianae*.

We further investigated the host range of the pathogen FQ01. The results showed that the pathogen FQ01 could infect tobacco leaves, tomato fruit, cucumber, eggplant fruit and potato tuber (Figure 1E), indicating that a variety of plants could be infected by the pathogen FQ01, especially postharvest fruit.

### Screening the antagonistic yeast strains for biocontrol of *P. nicotianae* FQ01

**Figure 2:**
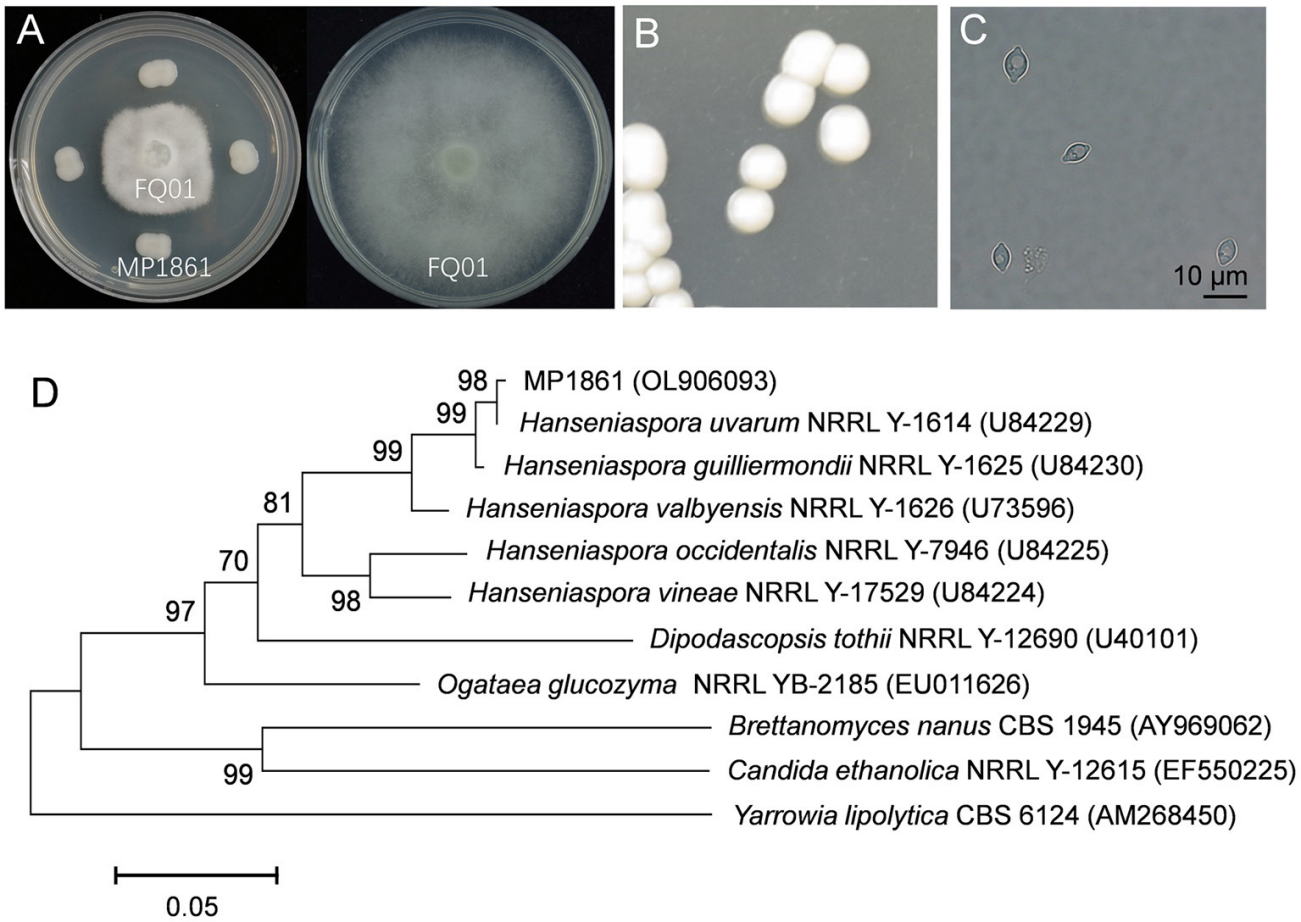
Identification of the antagonistic yeast strain MP1861. **(A)** Inhibition of *Phytophthora* growth by the yeast strain MP1861. **(B)** Colonies of the yeast strain MP1861 after growth on YPD plates at 26°C for 2 d. **(C)** Cell morphological characteristics of the yeast strain MP1861. **(D)** Phylogenetic relationship of the yeast strain MP1861 and other yeasts according to a neighbor-joining analysis of D1/D2 26S rDNA region.

We first isolated and purified more than 50 yeast strains from different plant samples. After testing the inhibitory activities of the isolated yeast strains against *P. nicotianae* FQ01, one candidate, labeled MP1861, exhibited the best biocontrol ability against the pathogen FQ01. As shown in Figure 2A, the growth of *P. nicotianae* FQ01 was strongly inhibited by the yeast strain MP1861.

To identify the yeast strain MP1861, the colony and cell morphological characteristics were investigated. As shown in Figure 2B, the colonies of the yeast strain MP1861 were white, round, smooth, and sticky. The cells of the yeast strain MP1861 exhibited a typical lemon shape (Figure 2C). A dendrogram based on the D1/D2 26S rDNA region of the yeast strain MP1861 (GenBank accession No. OL906093) was further constructed. According to the results of the phylogenetic analysis, the yeast strain MP1861 was identified as *H. uvarum* (Figure 2D).

### Inhibition of *P. nicotianae* FQ01 growth by the VOCs from *H. uvarum* MP1861

**Figure 3:**
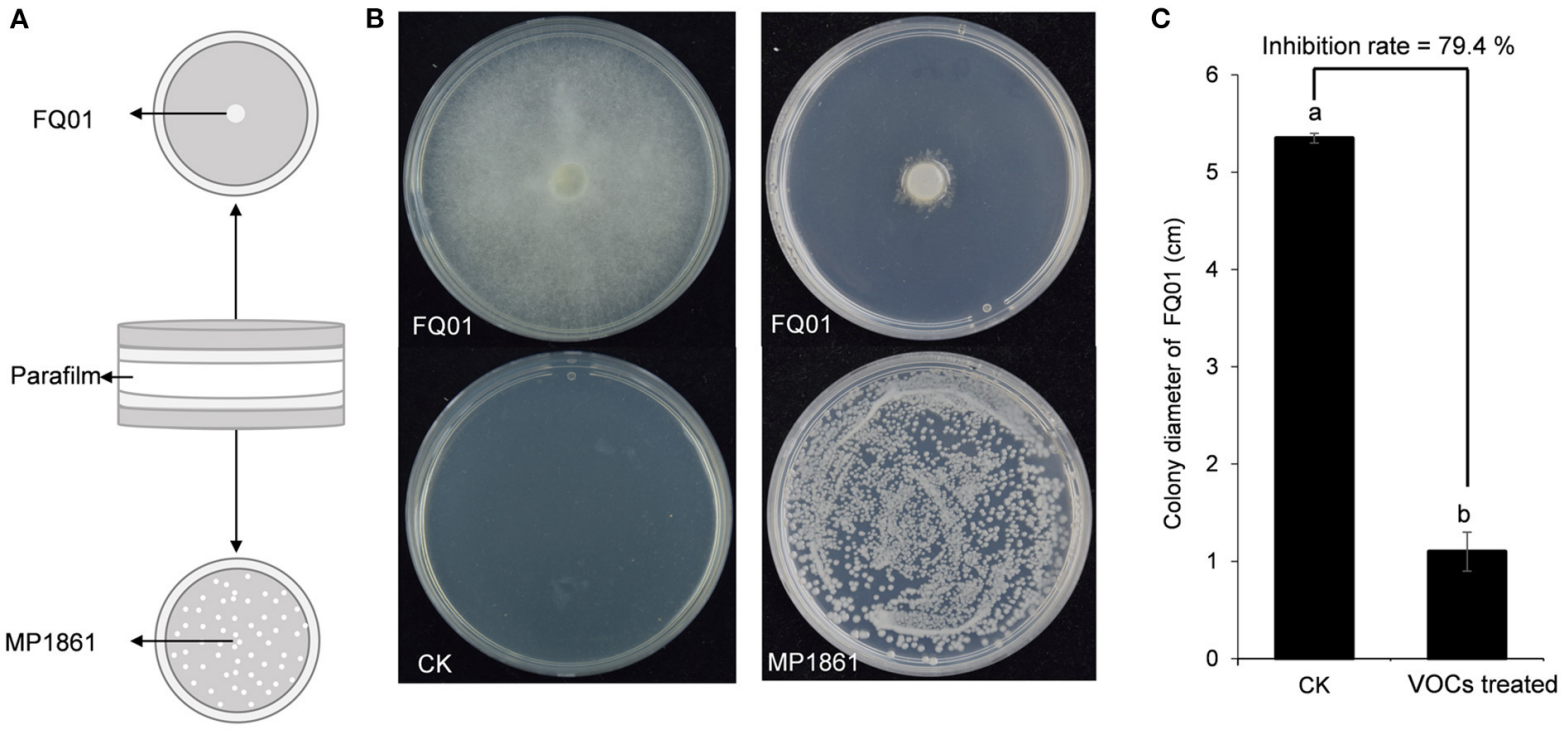
Graphical representation of VOCs released by the yeast strain MP1861 against the oomycete strain FQ01. **(A)** Schematic depiction of the method used in this study. **(B)** Mycelial growth of the pathogen FQ01 grown on PDA plates for 4 d. For the VOCs treated group, each plate was inoculated with 100 μl of yeast seed (3.0 ×10^4^ cells/mL). **(C)** Colony diameter of the pathogen FQ01. Data are calculated as the mean values ± SD, *n* = 3. The different letters a and b on the chart indicate significant difference at the 0.05 level.

A schematic representation for testing the inhibition of *P. nicotianae* FQ01 growth by the VOCs from *H. uvarum* MP1861 was shown in Figure 3A. The VOCs emitted by *H. uvarum* MP1861 could effectively inhibit the mycelial growth of *P. nicotianae* FQ01 (Figure 3B). The average colony diameter of the pathogen FQ01 reached 5.35 mm in the negative control treatment group and 1.10 mm in the antagonistic yeast treatment group (Figure 3C). The VOCs from *H. uvarum* MP1861 showed a growth inhibition rate of 79.4 % against *P. nicotianae* FQ01 (Figure 3C).

### Identification of the VOCs emitted by *H. uvarum* MP1861

**Figure 4:**
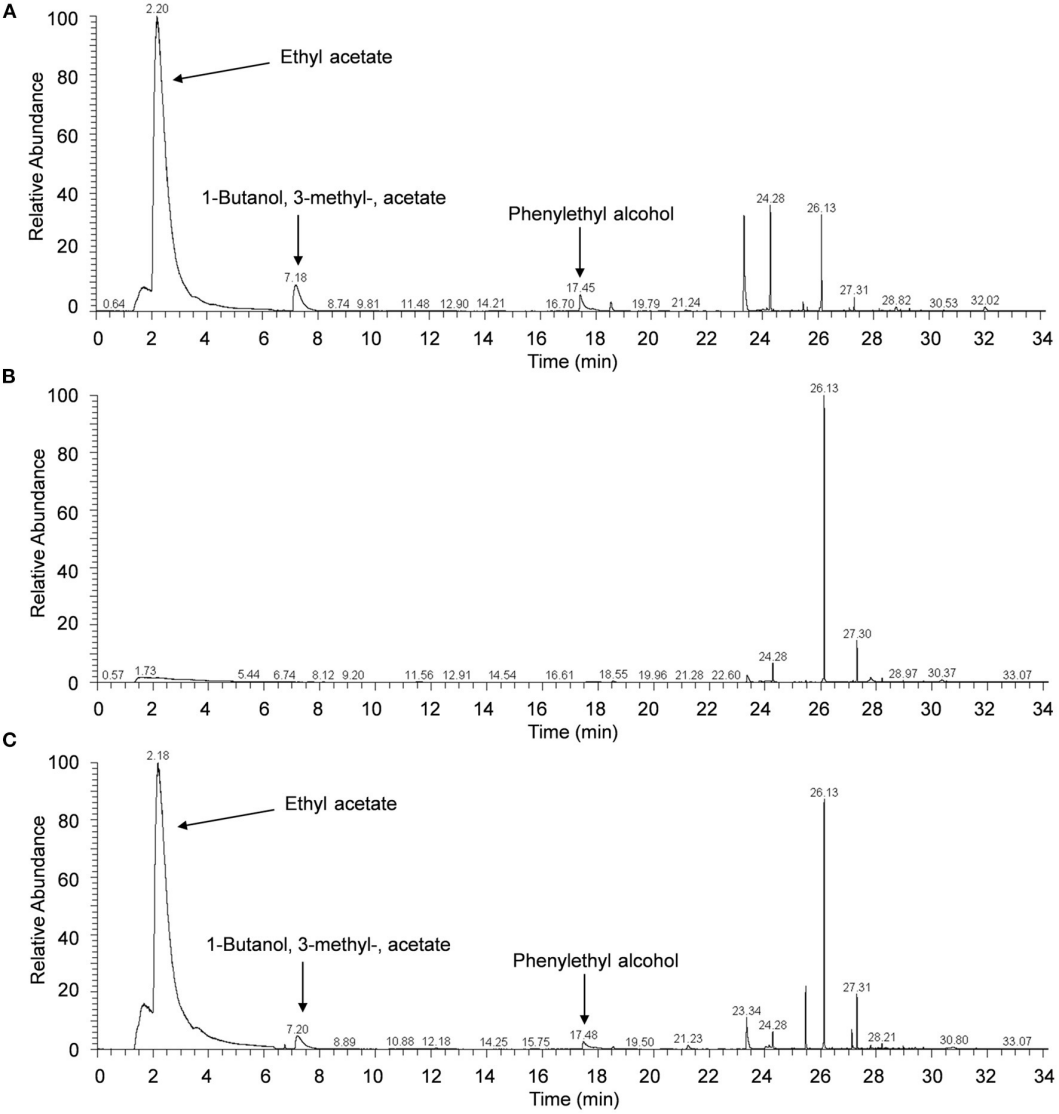
Chromatograms of VOCs. **(A)** The VOCs released from *H. uvarum* MP1861. **(B)** The VOCs produced by *P. nicotianae* FQ01. **(C)** The VOCs produced by coculture of the yeast and oomycete.

Results of GC/MS analysis showed that seven major components were identified in the VOCs released from *H. uvarum* MP1861, and their retention times were 2.20, 7.18, 17.45, 23.33, 24.28, 26.13, and 27.31 min (Figure 4A). However, only three components matched with more than 80% probability against the mass spectrometry database: ethyl acetate (2.20 min, probability 93.53%), 1-butanol, 3-methyl-, acetate (7.18 min, probability 83.47%) and phenylethyl alcohol (17.45 min, probability 86.31%) (Figure 4A). The MS spectra of these peaks (retention time: 2.20, 7.18, and 17.45 min) have been supplied in Supplementary Figure S1. It is worth noting that ethyl acetate was the most abundant component among the volatile gases detected above, accounting for 70.8% (Figure 4A). When the yeast strain MP1861 and the oomycete strain FQ01 were coincubated for 2 days, the main component among the VOCs was still ethyl acetate (Figure 4C). However, ethyl acetate was not detected in the VOCs produced by *P. nicotianae* FQ01 (Figure 4B).

### Confirmation of the growth inhibition of *P. nicotianae* FQ01 by ethyl acetate

**Figure 5:**
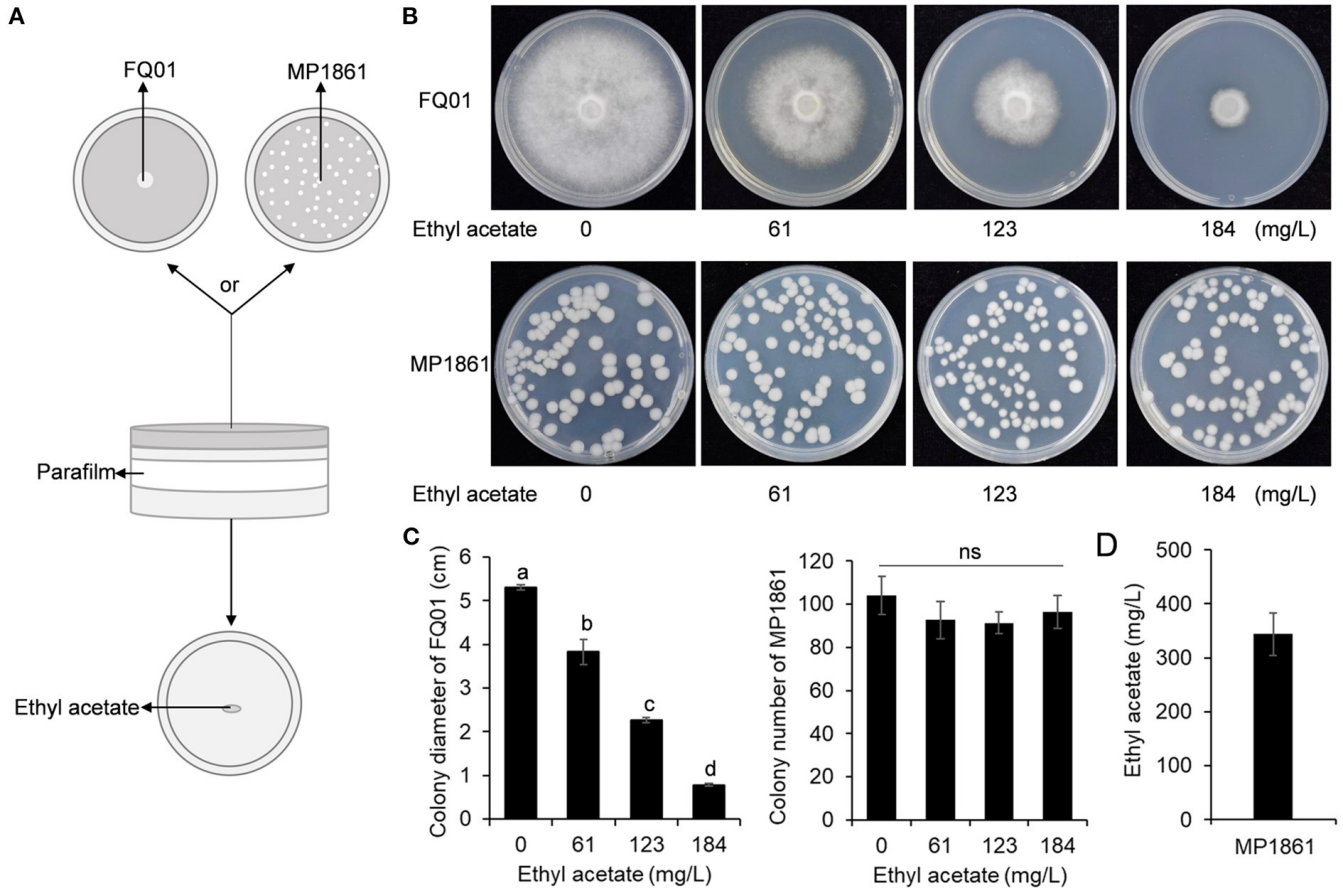
Antifungal activity of ethyl acetate in the airspace against *P. nicotianae* FQ01. **(A)** Procedure used in this study. **(B)** Colonies of the oomycete strain FQ01 and the yeast strain MP1861 grown on PDA plates for 4 d. **(C)** Colony diameter of the pathogen FQ01 and colony number of the yeast strain MP1861. Three experiments were carried out. Different letters (a, b, c, and d) labeled on the columns are significant difference at *p* < 0.05. Probability values of more than 0.05 were marked as ns (no significance). **(D)** Ethyl acetate production in the VOCs of the yeast strain MP1861.

Since ethyl acetate was the main component of the VOCs produced by the yeast strain MP1861 (Figure 4A), the impact of ethyl acetate on the growth of *P. nicotianae* FQ01 was further investigated. The procedure used in this study is shown in Figure 5A. The results revealed that with the increase in ethyl acetate content in the Petri dish airspace, the mycelial growth of the oomycete strain FQ01 was gradually inhibited. When the ethyl acetate content in the Petri dish airspace reached 184 mg/L, the mycelial growth of the oomycete strain FQ01 was completely inhibited (Figures 5B,C). However, the ethyl acetate concentrations tested in this study did not show inhibitory effect on the growth of the yeast strain MP1861 (Figures 5B,C). The ethyl acetate concentration in the VOCs released by the yeast strain MP1861 reached 344 mg/L (Figure 5D), which exceeded the tolerance of the oomycete strain FQ01 to ethyl acetate (Figures 5B,C).

### Plasma membrane damage of *P. nicotianae* by ethyl acetate

**Figure 6:**
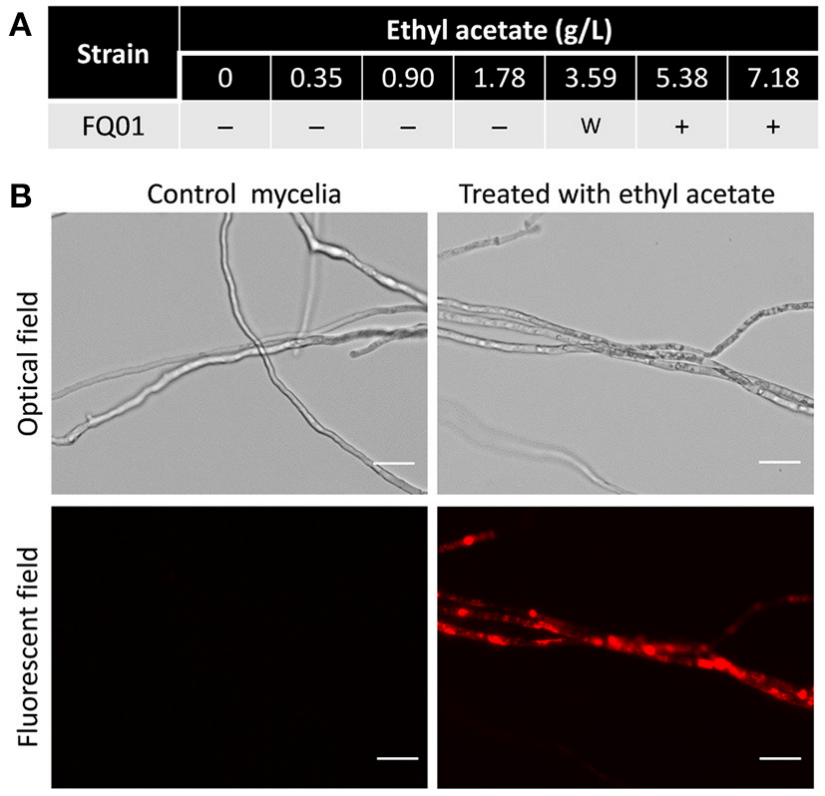
PI staining of the pathogen FQ01. **(A)** Effect of different ethyl acetate concentration on PI staining. –, negative; w, weak; +, positive. **(B)** Images of the pathogen FQ01 after PI staining. Mycelia treated with 5.38 g/L ethyl acetate. Bars represent 20 μm.

The toxicity of ethyl acetate to *P. nicotianae* FQ01 was investigated and the results showed that the mycelia of the pathogen FQ01 could not be stained by PI after being treated with low dose (<3.59 g/L) of ethyl acetate (Figure 6A), indicating that low dose of ethyl acetate showed weak toxicity to *P. nicotianae* FQ01. However, a strong red color was observed in the mycelia treated with 5.38 g/L ethyl acetate (Figure 6B), indicating that the plasma membrane integrity of the oomycete strain FQ01 was damaged by ethyl acetate.

### Effects of *H. uvarum* MP1861 VOCs on control of tomato fruit rot caused by *P. nicotianae* FQ01

**Figure 7:**
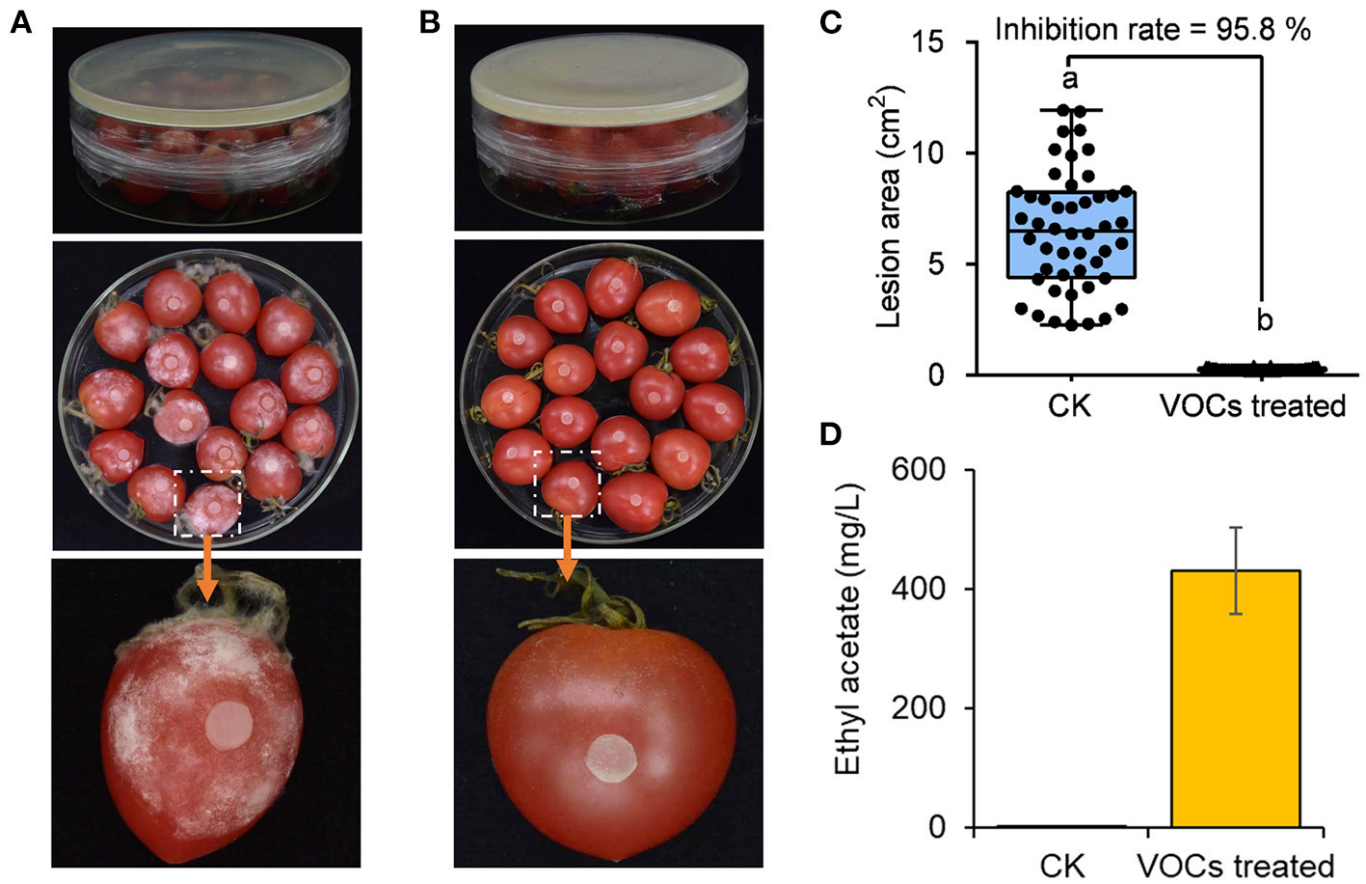
Evaluation of the efficacy of the VOCs from the yeast strain MP1861 on *Phytophthora* disease development. **(A)** Blank control group. **(B)** Group treated with *H. uvarum* MP1861 VOCs. **(C)** Statistics of the areas of 48 lesions on tomato fruit. Different letters, a and b, means a significant difference at the level of *p* < 0.05. **(D)** Ethyl acetate contents in the closed Petri dishes. Data are given as the mean values ± SD, *n* = 3.

The ability of the VOCs from *H. uvarum* MP1861 to suppress the pathogen FQ01-mediated disease development on tomato fruit was investigated. As shown in Figure 7A, all the tomato fruit inoculated with the oomycete strain FQ01 in the Petri plate showed severe symptoms of *Phytophthora* disease in the blank control group. Compared with those in the CK group, the lesions in the tomato fruit after biofumigation with the VOCs of *H. uvarum* MP1861 were basically confined to the inoculation area (Figure 7B). The inhibition rate of the VOCs on tomato fruit disease were 95.8% (Figure 7C). The concentration of ethyl acetate in the Petri plates containing *H. uvarum* MP1861 reached 430 mg/L (Figure 7D), indicating that the VOCs produced by the yeast strain MP1861 could protect tomato from *Phytophthora* pathogen infection.

## Discussion

Species of the genus *Phytophthora* are considered plant destroyers that can cause widespread damage in many agricultural species and even in native ecosystems worldwide. The established methods for effective control of the plant diseases caused by *Phytophthora* rely mainly on extensive use of fungicides (Panabieres et al., 2016). However, in terms of food safety and environmental friendliness, these methods are no longer suitable given the current situation. In recent years, the demand for safe agricultural products has increased. Therefore, the utilization of biological control agents as replacements for chemical pesticides has attracted great interest. Numerous previous researches have indicated that selected strains of many antagonistic bacteria and filamentous fungi, including *B. atrophaeus* (Rajaofera et al., 2018), *P. fluorescens* (Sukhada et al., 2011), *G. mosseae* (Sukhada et al., 2011) and *Trichoderma* spp. (Bae et al., 2016), were effective agents for biocontrol of plant diseases caused by *P. nicotianae*. Our study revealed that antagonistic yeast was also an important source for screening biocontrol agent against *P. nicotianae* similar to antagonistic bacteria and filamentous fungi.

A large number of studies have shown that the biological control mechanisms of antagonistic yeasts against plant pathogens include competition for nutrients and space (Zhao et al., 2008; Zhang et al., 2011; Cheng et al., 2019), secretion of extracellular cell wall-degrading enzymes (Chanchaichaovivat et al., 2008; Zhang et al., 2011), restriction of spore germination and germ tube growth of pathogens (Droby et al., 2002), induction of disease resistance in host plants (Droby et al., 2002; Zhao et al., 2008; Apaliya et al., 2017), etc. In this study, it was found that the VOCs produced by *H. uvarum* MP1861 could effectively suppress the mycelial growth of *P. nicotianae* FQ01. Consistent with our observations, production of VOCs with antifungal activities have been reported in various yeasts e.g., *Candida intermedia* (Huang et al., 2011), *Hanseniaspora osmophila* (Delgado et al., 2021), *Starmerella bacillaris* (Lemos Junior et al., 2020), *Wickerhamomyces anomalus, Metschnikowia pulcherrima* and *Saccharomyces cerevisiae* (Oro et al., 2018). In another report, the VOCs produced by *H. uvarum* reduced *B. cinerea* infection of strawberry (Qin et al., 2017). These findings suggest that biosynthesis of antifungal VOCs by antagonistic yeasts is one of the important mechanisms for controlling plant pathogens.

VOCs are substances with a low molecular weight (<300 Da), high vapor pressure (≥0.01 kPa at 20°C), and low water solubility (Pagans et al., 2006; Delgado et al., 2021). The VOCs synthesized by microbes contains a variety of compounds such as esters, alcohols and aromatic compounds (de Boer et al., 2019). The present study revealed that the volatiles produced by *H. uvarum* MP1861 contained ethyl acetate, isopentyl alcohol (1-butanol, 3-methyl-, acetate), and phenylethyl alcohol. Similarly, these volatiles were also detected in *C. intermedia* C410 (Huang et al., 2011) and *H. osmophila* 337 (Delgado et al., 2021). According to previous researches (Huang et al., 2011; Qin et al., 2017), these volatiles have been demonstrated antifungal activities. Compared with the yeast strains reported above, the yeast strain MP1861 had the highest content (70.8 %) of ethyl acetate in the VOCs. Our study revealed that ethyl acetate was the main antifungal component in the VOCs of *H. uvarum* MP1861.

*P. nicotianae* was found to be sensitive to both esters and oils. It was found that garlic essential oil could strongly inhibit the growth of *P. nicotianae*, and one of the major components in garlic essential oil is diallyl disulfide, which could destroy the cell membrane of *Phytophthora* and cause cell death (Wang Y. C. et al., 2019). The cell membrane integrity of *P. nicotianae* could also be destroyed by essential oil from *Chrysanthemum indicum* L., in which the major components are monoterpenes and sesquiterpenes (Han et al., 2019). In another study, eugenol, a major component in *Syringa oblata* essential oil, inhibited the mycelial growth of *Phytophthora* by impairing its cell membrane (Jing et al., 2017). We found that ethyl acetate also destroyed the cell membrane of *P. nicotianae*, leading to cell death. These observations confirmed the inhibitory effects of oils and esters on *P. nicotianae*.

Ethyl acetate has been widely used in food, beverage, solvent and other fields (Zhang et al., 2020). However, ethyl acetate isn't 100% safe. It can cause heat/sparks/open flames/hot surfaces. Exposure to ethyl acetate even at low concentration causes dizziness, irritation and even cancer (Zhu et al., 2017). In addition, this component can be toxic if ingested or inhaled and can cause severe internal organ damage with long-term or repeated exposure. Control of plant diseases using the antagonistic yeast strain MP1861 can avoid the potential harm caused by direct application of ethyl acetate in the environment. Biological control of plant disease using antagonistic yeast involves a tritrophic interaction between host, pathogen and yeast, all of which are influenced by environmental factors such as temperature, pH, ultraviolet light, nutrition, and osmotic and oxidative stress (Sui et al., 2015). The influence of environmental factors on the production of ethyl acetate by *H. uvarum* MP1861 will be further investigated.

## Conclusion

The current work demonstrated that the VOCs released by *H. uvarum* MP1861 exhibited bioactivity against *P. nicotianae* FQ01. Ethyl acetate was identified to be the most abundant component in the VOCs produced by the yeast strain MP1861, which exhibited strong anti-oomycete activity against *P. nicotianae* FQ01 by breaking the *Phytophthora* cell membrane integrity. The yeast strain MP1861 significantly decreased the incidence of decay on tomato fruit caused by *P. nicotianae*. This study provided a novel antagonistic yeast strain MP1861 of *H. uvarum* and a useful information about the antagonistic properties of *H. uvarum* MP1861. Further studies are warranted to focus on the application of *H. uvarum* MP1861 in the control of *Phytophthora* diseases in agricultural production.

## Data availability statement

The original contributions presented in the study are included in the article/Supplementary material, further inquiries can be directed to the corresponding author/s.

## Author contributions

ZL and GW designed the experiments and wrote the first draft of the manuscript. ZL and JT performed the experiments. ZL, JT, HY, and XW organized the database and performed the statistical analysis. DL, GW, and WL reviewed and edited the manuscripts. All authors provided comments on the manuscript and approved the submitted version.

## Funding

This work was financially supported by the Shandong Provincial Natural Science Foundation (ZR2019MC052, ZR2020KC003), the National Natural Science Foundation of China (31972213), the Key Research and Development Program of Shandong Province (2019YQ017), Shandong Province Double-Hundred Talent Plan (WST2018008), and Taishan Scholar Construction Foundation of Shandong Province (tshw20130963).

## Conflict of interest

The authors declare that the research was conducted in the absence of any commercial or financial relationships that could be construed as a potential conflict of interest.

## Publisher's note

All claims expressed in this article are solely those of the authors and do not necessarily represent those of their affiliated organizations, or those of the publisher, the editors and the reviewers. Any product that may be evaluated in this article, or claim that may be made by its manufacturer, is not guaranteed or endorsed by the publisher.

## Supplementary material

Supplementary Figure S1 MS spectra of three peaks. **(A)** Ethyl acetate peak at 2.20 min. **(B)** 1-butanol, 3-methyl-, acetate peak at 7.18 min. **(C)** Phenylethyl alcohol peak at 17.45 min.
