# Supplementary figures and images for: Ethyl acetate produced by Hanseniaspora uvarum is a potential biocontrol agent against tomato fruit rot caused by Phytophthora nicotianae
Source: Front Microbiol. 2022 Aug 10;13:978920. doi: 10.3389/fmicb.2022.978920 (PMC9399722; doi:10.3389/fmicb.2022.978920)

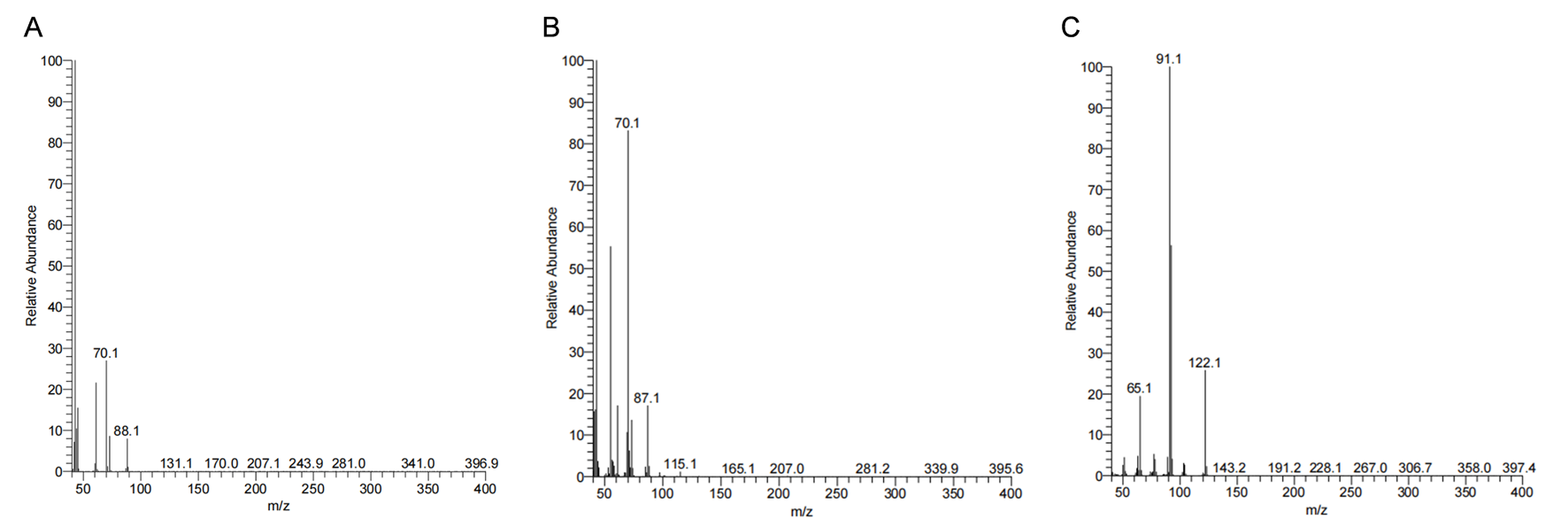

Supplement: Supplementary Figure S1 — MS spectra of three peaks. (A) Ethyl acetate peak at 2.20 min. (B) 1-butanol, 3-methyl-, acetate peak at 7.18 min. (C) Phenylethyl alcohol peak at 17.45 min. [file Image_1.TIF]
